# Selective patterning of ZnO nanorods on silicon substrates using nanoimprint lithography

**DOI:** 10.1186/1556-276X-6-159

**Published:** 2011-02-21

**Authors:** Mi-Hee Jung, Hyoyoung Lee

**Affiliations:** 1 Thin Film Solar Cell Technology Research Team, Advanced Solar Technology Research Department, Convergence Components & Materials Research Laboratory, Electronics and Telecommunications Research Institute, Daejeon, Republic of Korea; 2 National Creative Research Initiative, Center for Smart Molecular Memory, Department of Chemistry, Sungkyunkwan University, 300 Cheoncheon-dong, Jangan-gu, Suwon 440-746, Republic of Korea

## Abstract

In this research, nanoimprint lithography (NIL) was used for patterning crystalline zinc oxide (ZnO) nanorods on the silicon substrate. To fabricate nano-patterned ZnO nanorods, patterning of an *n*-octadecyltrichlorosilane (OTS) self-assembled monolayers (SAMs) on SiO_2 _substrate was prepared by the polymer mask using NI. The ZnO seed layer was selectively coated only on the hydrophilic SiO_2 _surface, not on the hydrophobic OTS SAMs surface. The substrate patterned with the ZnO seed layer was treated with the oxygen plasma to oxidize the silicon surface. It was found that the nucleation and initial growth of the crystalline ZnO were proceeded only on the ZnO seed layer, not on the silicon oxide surface. ZnO photoluminescence spectra showed that ZnO nanorods grown from the seed layer treated with plasma showed lower intensity than those untreated with plasma at 378 nm, but higher intensity at 605 nm. It is indicated that the seed layer treated with plasma produced ZnO nanorods that had a more oxygen vacancy than those grown from seed layer untreated with plasma. Since the oxygen vacancies on ZnO nanorods serve as strong binding sites for absorption of various organic and inorganic molecules. Consequently, a nano-patterning of the crystalline ZnO nanorods grown from the seed layer treated with plasma may give the versatile applications for the electronics devices.

## Introduction

Zinc oxide (ZnO) nanorods have been widely investigated in applications such as ultraviolet nanolaser sources, gas sensors, solar cells, and field emission display devices because they have a direct band gap of 3.37 eV and a large exciton binding energy of 60 meV. As various applications of nanostructured materials, it is very important not only to synthesize the ZnO nanorods with a high degree of regularity and uniformity in terms of diameter and length, but also to accurately position them in arrays.

Traditionally, the aligned growth of ZnO nanorods has been successfully achieved on solid substrates via a vapor-liquid-solid (VLS) process with the use of gold and tin as catalysts [1]. The VLS process may risk introducing catalyst residual atoms into the ZnO nanorods, which is incompatible with silicon technology and additionally requires heat treatment at high temperatures, which can damage substances already present on the substrates. As a result, the patterned growth of aligned ZnO nanorods has been conducted on expensive substrates, including GaN, SiC, and sapphire [2]. Thus mass production of high-quality-patterned ZnO nanorods at low cost is still a challenge.

Until now, wet chemical processing among the various methods is desirable due to the relatively low processing cost with merits of low growth temperature, economical synthesis, and good potential for scale-up when compared with the VLS method. Recently, a solution method for patterning the ZnO nanorods using the self-assembled monolayer (SAM) template was reported. Julia et al. [3] demonstrated the direct growth of the ZnO nanorods on silver films from aqueous solution using the organic template because the ZnO nucleation was inhibited through appropriate complexation with the carboxylate end groups of ω-alkanethiol SAM molecules on the silver substrate. Koumoto et al. [4] reported that pre-patterned SAMs of a hydrophobic end group led effectively to pattern a ZnO nanocrystal.

Nanoimprint lithography (NIL) has a high throughput and low-cost process and is well-suited for mass production [5,6]. NIL is immune to the many factors that limit conventional photolithography resolution, such as diffraction, scattering, and the interference in the resist, backscattering from a substrate, and the developer chemistry. This method allows the patterning of three dimensions, with feature sizes down to 6 nm [7]. Due to these advantages, NIL was used for the direct patterning of functional semiconducting organic material [8], molecular electronic devices [9], and fabrication of nanowire [10].

Here, we presented new patterning methods of ZnO nanorods which were combined with the selective patterned ZnO seed layer, surface polarity, and NIL. The ZnO seed layer was made by SAM patterns which were prepared by the polymer mask pattern which was made by NIL [5-7,11-13]. The polymer mask was used to create the nano-patterned OTS SAMs which can be used for patterning the ZnO seed layer. After lift-off of the polymer mask, the patterns of the ZnO seed layer were selectively produced on the site where the polymer mask was removed, not on the OTS SAMs. After the annealing process of the ZnO seed layer, the substrate was treated with an oxygen plasma to remove the SAMs and simultaneously increase the surface polarity of the ZnO seed layer. As a result, ZnO growth was only allowed on the ZnO seed layer due to the enhanced surface polarity. Therefore, we expect that the selective patterning of the ZnO nanorods can be obtained with mass production at low temperature without the use of catalysts.

## Experimental section

### SAM formation

The densely packed OTS SAMs were formed by soaking in a 5 mmol hexadecane:chloroform (4:1 vol.%) solution of OTS in an N2 glove box for 3 h. After baking at 120°C for 10 min, a siloxane bond was formed on the silicon oxide surface.

### Nanoimprint lithography

The nanoimprint processes were performed using an IMPRIO 100 (Molecular Imprint Inc., MII). Poly(methyl-methacrylate) (PMMA) (950K, A2) used as a planarization layer was spin-coated with a thickness of 65 nm on the Si (100) substrate. The first layer of the double resists, PMMA, was served not only as a planar layer, but also a residual layer after the imprint process. Hence, we treated the PMMA surface with oxygen plasma to decrease the thickness of PMMA to 25 nm. The treatment of the PMMA surface with oxygen plasma was also changed to the hydrophilic surface, resulting in an increased adhesion force between the PMMA surface and the second layer, a Si containing resin. The Si containing acrylate-based monomer mixture supplied by MII was dispensed on the PMMA surface in a drop-on-demand manner using a jet-type dispenser. The quartz mold was lowered to a surface dispensed with a resin until the resin completely filled the mold geometry. After the resist was cured via exposure to UV (λ = 365 nm) light, the mold was separated from the cured resist. Finally, the PMMA residual layer in the compressed area was removed via anisotropic reactive ion etching, thereby exposing the substrate surface.

### ZnO growth

A substrate was spin-coated with a droplet of 0.01 M zinc acetate dihydrate aqueous solution at 5000 rpm for 30 s. The spin-coating process was repeated three to four times to insure uniform coating on the entire substrate. The substrate coated with a film of zinc acetate crystallites was heated to 300-500°C in air for 1 h to yield the ZnO seed layer. The surface coated with the ZnO seed layer was exposed to a solution containing 50 mM equimolar concentration of hexamethylenetetramine (HMT) and zinc nitrate at 95°C for 10 min to 3 h.

### Characterizations

SEM images of the ZnO nanorods were obtained with a field emission scanning electron microscope (FEI, Model: Sirion, Netherlands). X-ray diffraction (XRD) patterns were measured by using a D8 Discover thin-film diffractometer with Cu Kα radiation (40 kV, 30 mA, λ = 1.54056 Å) and a Ni filter plus a graphite monochromator. The XRD spectrum was recorded in a Bragg-Brentano configuration using θ/2θ scanning and no tilt angle. Photoluminescence (PL) measurements on the ZnO arrays were performed using the 325 nm line of a He-Cd laser as the excitation source at room temperature. Atomic force microscopy (AFM) measurements were carried out on a Multimode XE-100 instrument (PSIA Inc.) operating in without contact with silicon cantilevers (resonance frequency in the range 204-259 kHz, an integrated Si tip with a typical radius of 10 nm curvature) and with contact with silicon nitride cantilevers (resonance frequency 56 kHz).

## Results and discussion

**Figure 1:**
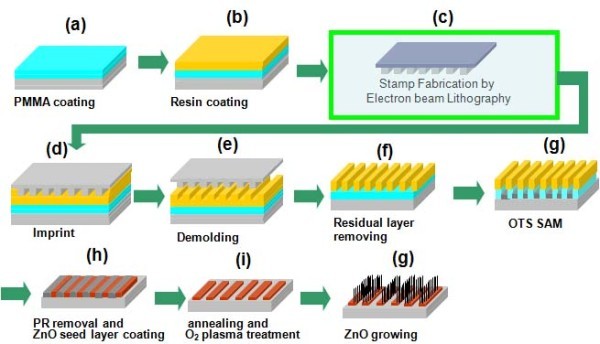
**Schematic diagram depiction of the ZnO patterning process using the polymer template formed by nanoimprint lithography**: **(a) **PMMA coating, **(b) **resin coating pattern, **(c) **stamp fabrication, **(d) **imprint process, **(e) **demolding, **(f) **residual layer removing, **(g) **OTS self assembly monolayer, **(h) **PR removal and ZnO seed layer coating, **(i) **annealing and O_2 _plasma treatment, and **(j) **ZnO growing.

**Figure 2:**
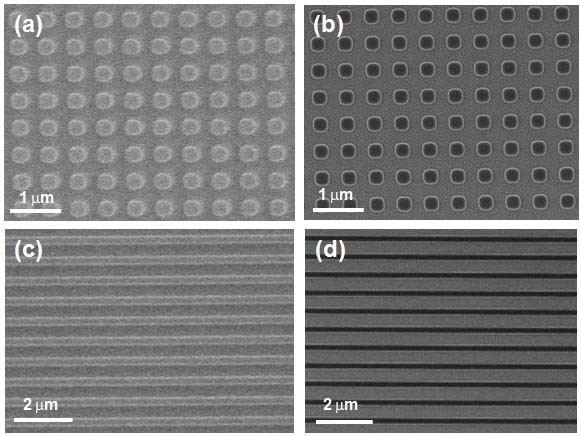
**SEM images of nanopattern from NIL results on the SiO_2_/Si substrates including a square lattice of circular pillars of 300 nm diameter with a 200 nm pitch**. before **(a) **and after **(b) **reactive ion etching, lines of 300 nm width with a pitch of 200 nm before **(c) **and after **(d) **reactive ion etching.

**Figure 3:**
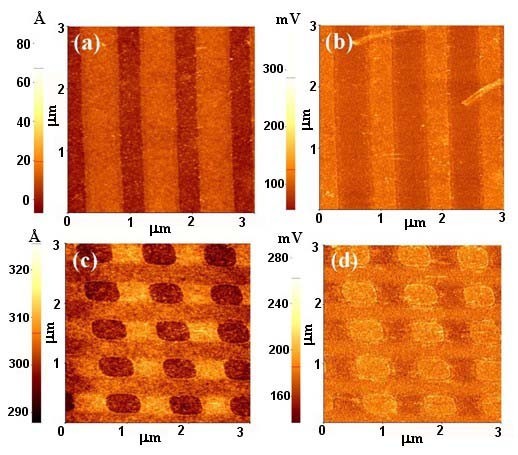
**AFM images of the OTS SAM surfaces**. **(a, c) **Topographic images of OTS SAMs. **(b, d) **AFM images with lateral force mode on the structured SAMs. The darker regions correspond to lower friction areas consisting of hydrophobic silane SAM.

Figure 1 showed the scheme of ZnO patterning process on a silicon surface. To fabricate the nanopatterned ZnO nanorods, we used a polymer mask which was made by NIL. Figure 2 showed SEM images of nanopattern obtained from NIL results on the SiO_2_/Si substrates, giving a square lattice of circular pillars of 300 nm diameter with a 200 nm pitch. Figure 2a,b depicted before and after reactive ion etching, respectively. Figure 2c,d showed lines of 300 nm width with a pitch of 200 nm before and after reactive ion etching, respectively. As can be seen in Figure 2, the imprint process can reproducibly print nanostructures with high fidelity over large area. The thickness of the polymer nanostructures was dependent on the height of the molds. The polymer islands were acted as masks. The polymer mask was then subjected to a brief oxygen plasma step to remove the intermediate layer between the bumps and expose the silicon oxide surface. The power and duration of the plasma exposure were chosen in such a way as to result in the removal of the continuous thin intermediate layer of the polymer. The subsequent exposure to OTS vapors resulted in selective silanization only on the exposed silicon oxide region, giving OTS SAMs. After lift-off of the polymer mask, the surfaces of the patterned SAMs were evaluated by AFM with contact and lateral force microscopy (LFM), respectively. Figure 3a,c presented the topographies of a binary pattern consisting of the methyl (-CH_3_) of OTS SAMs and hydroxyl (-OH) terminal group of SiO_2 _surface. Figure 3b,d showed that the SiO_2 _surface terminated with -OH group produced the regions of high friction due to the strong interactions between the AFM tip and the surface while the AFM tip experienced low torsion in the regions of OTS terminated by the methyl functional group. This difference yielded a strong contrast when imaged by LFM [14,15].

The polymer masks were lifted off by acetone immersion to produce the nanopatterned SAMs. The seed layer, 0.01 M zinc acetate dihydrate dissolved in ethanol, was spin-coated on the patterned SAM surface at 5000 rpm for 30 s and followed by drying at 70°C on a hot plate to remove the ethanol solvent. This process was repeated three or four times to obtain a uniform Zn(OAc)_2 _film. The ZnO seed layer was selectively coated only on the SiO_2 _surface terminated with -OH, not on the SAMs terminated with -CH_3 _group. The patterned array substrate was annealed at 300-500°C for 1 h to change the Zn(OAc)_2 _into the crystalline ZnO [16,17]. After the annealing process, a surface coated with the ZnO seed layer was treated by the oxygen plasma to enhance the surface polarity and simultaneously to remove the OTS SAMs. The oxygen plasma can promote the -O_*n*_Si(OH)_4-__*n *_group on the silicon surface, which is highly dependent on the intensity of oxygen plasma and the treatment time [18]. The treatment of the oxygen plasma yielded a narrow nucleation region in which the oxidation state transition was passivated from fully oxidized to partially oxidized or non-oxidized state while it removed organic residues on the SiO_2 _surface, which is required to accomplish uniform growth over the entire wafers. Thus, treatment with strong plasma for a long time could induce the ZnO seed layer to oxidation state that was too strong to grow ZnO from the seed layer. Control experiments indicated that the plasma treatment of the ZnO seed layer generated the best results with O_2 _at a pressure of 30 mTorr, flow rate of 20 sccm, and power intensity of 50 W (Oxford Plasma Lab 100 ICP (380 etcher)) for 2 min. Previous studies showed that the enhanced surface polarity was used for ZnO crystal growth. Lee et al. reported the fabrication of ordered arrays of ZnO nanorods using controlled polar surfaces of ZnO templates [19]. Jacobs et al. [20] used the oxygen plasma and photoresist patterns to produce ZnO single-crystal structures of high quality on the Mg-doped GaN substrates. The oxygen plasma was used to oxidize Mg dopant to inhibit ZnO nucleation and to nucleate ZnO growth on the non-oxidized Mg sites.

**Figure 4:**
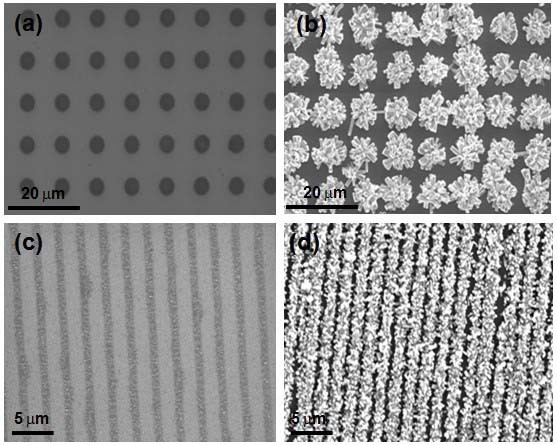
**SEM images**. **(a, c) **seed layer pattern and **(b, d) **ZnO pattern grown from the seed layer, respectively.

The plasma-treated surface coated with the ZnO seed layer was suspending the wafer facedown in an equimolar aqueous solution (0.1 M) of zinc nitrate hexahydrate [Zn(NO_3_)_2_⋅6H_2_O] and hexamethylenetetramine at 95°C to grow the ZnO nanorods via the hydrothermal method [21-23]. In a typical solution, the pH of the solution was kept at 6-7. It was found that the nucleation and initial growth of the crystalline ZnO were accelerated on the ZnO seed layer, not on the oxidized SiO_2 _surfaces. The plasma-treated silicon surface should suppress OH- attachment and nucleation toward the center of exposed ZnO regions. Therefore, ZnO nanorods can be grown only on Zn-polar seed layer surfaces [24-26] and the hexamethylenetetramine and amine-mediated additives, which are nonpolar chelating agents [27], would preferentially attach to the nonpolar facets, thereby exposing the polar planes (*c*-axis) for anisotropic growth. Considering the SiO_2 _point of zero charge, we speculate that ZnO nucleation on oxidized SiO_2 _location may be possible if growth pH is held above the pH 2. It is widely accepted as 2 for a zero-charged bulk SiO_2_. However, formation of an ionic Si-O bond through plasma oxidation deactivated nucleation. This implied that strongly oxidized SiO_2 _surface suppressed OH- attachment and preferentially allowed to nucleate ZnO only on the ZnO seed layer for the experimental pH ranges (pH 6-7) described here. Figure 4a,c showed SEM images of ZnO seed layer pattern on the SiO_2 _surface and Figure 4b,d depicted the ZnO nanorod array grown from the ZnO seed layer pattern.

**Figure 5:**
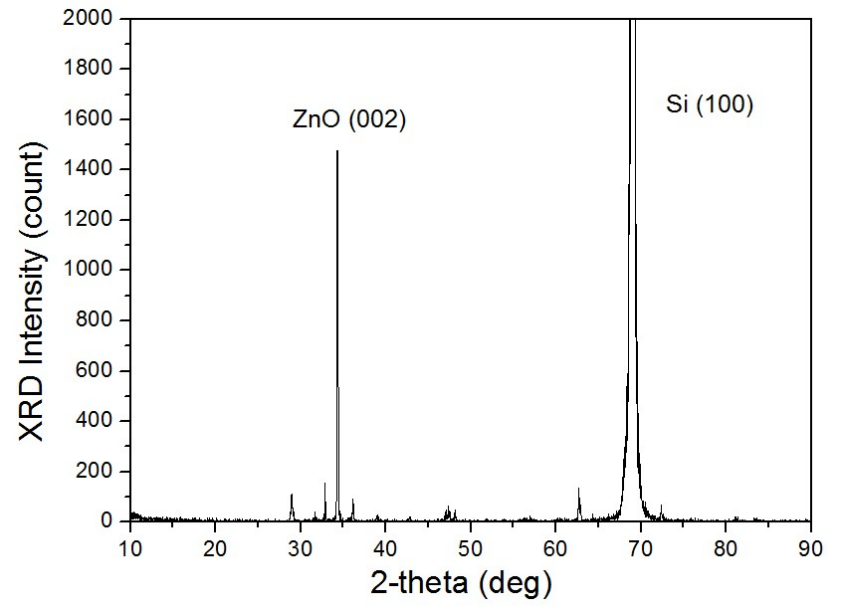
**X-ray diffraction patterns of the as-grown ZnO arrays on the plasma-treated seed layer**.

**Figure 6:**
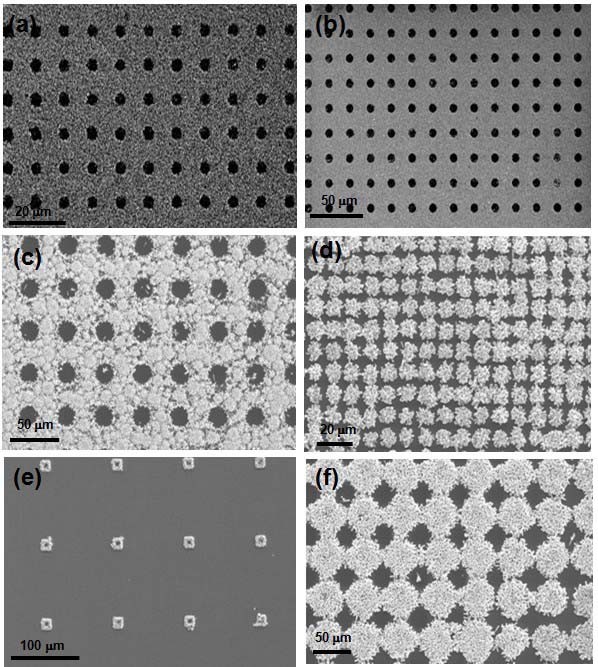
**SEM characterization of ZnO nanorods on silicon. Rod length and diameter are 250-400 and 60-80 nm, respectively**. **(a-c) **Negative template pattern, **(d-f) **positive template pattern.

The crystal structure and vertical alignment of the as-grown ZnO pattern were examined by XRD and rocking curve measurements. The θ-2θ scanning results of the sample were shown in Figure 6. XRD patterns as shown in Figure 5 showed strong peaks at 2θ = 34.393° attributed to the ZnO (002) crystal plane with a Wurtzite structure with lattice parameters *a *= 3.296 Å and *c *= 5.207 Å. It exhibited full width at half maximum value of 0.15°, which indicates almost perfect *c*-axis perpendicular alignment of the ZnO nanorods on the seed layer.

Figure 6a-c and 6d-f illustrated several patterned ZnO nanorod arrays fabricated by negative or positive templates, respectively. It is expected that our patterning process can be easily applied to synthesize ZnO nanorod arrays on other substrates such as transparent glass and flexible polymer substrates in an aqueous solution under ambient conditions.

**Figure 7:**
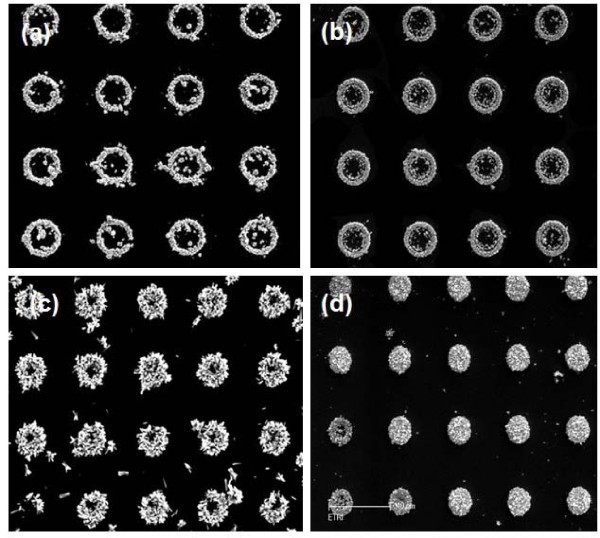
**ZnO nucleated and grown at the corner or edges of the seed layer**. The growth time is **(a) **15 min, **(b) **30 min, **(c) **1 h, and **(d) **2 h. The scale bar is 10 μm.

Since the corners or edges of the patterned structure were often the preferred nucleation sites for material deposition, the catalytic atoms may diffuse preferentially to the corner to form the ZnO nucleus. Figure 7 confirmed that the ZnO pattern was initially a ring pattern and toward the center from an edge or corner of a rectangle, the seed layer was completely filled with the ZnO nanorods within 2 h. The density and morphologies of ZnO nanorods on the seed layer were mainly determined by the solvent, precursor, acidity-basicity of solution, and reaction temperature as well as reaction time [28]. Here, when the size of the seed layer is enough small, only one ZnO nanorod was formed.

**Figure 8:**
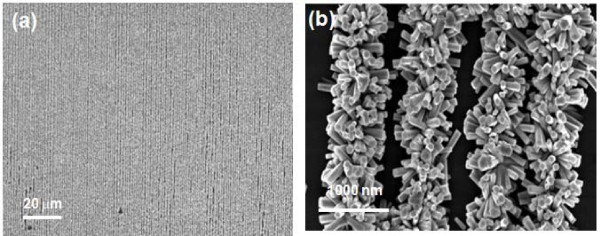
**SEM image of (a) ZnO nanoline pattern and (b) enlarged image of pattern (a)**.

For the array patterning, one of the main advantages of NIL technology is the ability to push the resolution to the nanometer scale with mass production. We fabricated the 800 nm nanolines of ZnO nanorods, starting from seed layer pattern on the silicon surface, as depicted in Figure 8.

**Figure 9:**
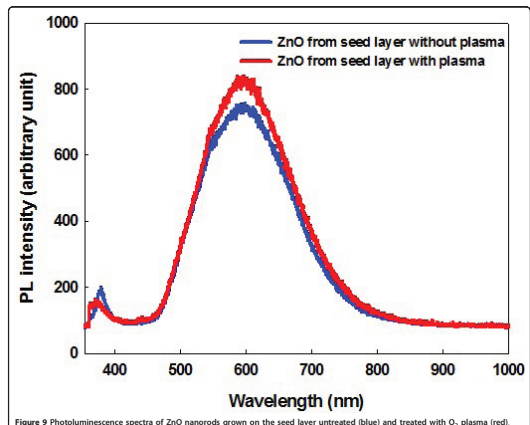
**Photoluminescence spectra of ZnO nanorods grown on the seed layer untreated (blue) and treated with O_2 _plasma (red)**.

The optical properties of the patterned ZnO arrays were obtained with PL measurement. Figure 9 showed the PL spectra of ZnO nanorods that were untreated or treated with oxygen plasma grown from seed layer, respectively. Grown from aqueous solution at room temperature, ZnO nanorods exhibited a weak band edge emission at 378 nm resulting from free-exciton annihilation [29] and a very strong and broad yellow-orange emission at 605 nm attributed to oxygen vacancy [30,31]. The difference of PL intensity provides the luminescent properties dependent on the surface polarity of the seed layer. The ZnO nanorods grown from the seed layer treated with plasma showed lower intensity than those grown from the seed layer untreated with plasma at 378 nm, but higher intensity at 605 nm. It is indicated that the seed layer treated with plasma produced ZnO nanorods that had a more oxygen vacancy than those grown from seed layer untreated with plasma. Surface defects of the ZnO nanorods such as oxygen vacancies allowed serving as strong binding sites for absorption of various organic and inorganic molecules [32]. Therefore, ZnO nanorods grown from the seed layer treated with plasma can be easily modified with several materials and controlled for the electronic properties, yielding a tunable application for the electronic devices.

## Conclusions

In conclusions, NIL was used for patterning crystalline ZnO nanorods on the silicon substrate. The seed layer was coated between the OTS SAMs, resulting in a selective coating only on the hydrophilic silanol surface. The substrate patterned with the seed layer was treated with the oxygen plasma to oxidize the silicon surface. It was found that the nucleation and initial growth of the crystalline ZnO were accelerated on the ZnO seed layer, not on the silicon oxide surfaces. The plasma-treated silicon surface should suppress OH- attachment and nucleation toward the center of exposed ZnO regions. The ZnO photoluminescence showed that the ZnO nanorods grown from the seed layer-treated plasma showed lower intensity than those grown from the seed layer-untreated plasma at the 378 nm, but higher intensity in the 605 nm. It is indicated that the seed layer treated with plasma produced the ZnO nanorods which had a more oxygen vacancy than those grown from the seed layer untreated with plasma. We fabricated the 800 nm nanolines with ZnO nanorods, starting from seed layer pattern on the silicon surface.

## Abbreviations

AFM: atomic force microscopy; HMT: hexamethylenetetramine; LFM: lateral force microscopy; NIL: nanoimprint lithography; PL: photoluminescence; PMMA: poly(methyl-methacrylate); SAM: self-assembled monolayer; VLS: vapor-liquid-solid; XRD: X-ray diffraction; ZnO: zinc oxide.

## Competing interests

The authors declare that they have no competing interests.

## Authors' contributions

MHJ carried out the ZnO patterning process with the nanoimprint lithography and drafted the manuscript. HL conceived of the study, and participated in its design and coordination.
